# Vegetarianism and Its Implications for Body Mass Index, Health, and Dietary Awareness in a Metropolitan City in India: A Cross-Sectional Study

**DOI:** 10.7759/cureus.42187

**Published:** 2023-07-20

**Authors:** Anjani H Turaga

**Affiliations:** 1 Medicine and Surgery, Gandhi Medical College, Hyderabad, IND

**Keywords:** lacto-ovo vegetarian, diet modification, diet, vegetarian dietary patterns, vegetarian diet

## Abstract

Background

This cross-sectional study aimed to assess and analyze the awareness of vegetarianism and its prevalence as a lifestyle practice in an urban population of Hyderabad, India.

Methodology

A predesigned, semi-structured questionnaire was used to collect data from consenting study participants. The questionnaire was administered, and the response data were extracted and analyzed using Microsoft Excel and Open Epi. The chi-square test was employed, and an appropriate p-value was set for each parametrical calculation and analysis.

Results

The study revealed that 43.7% of the study group followed a vegetarian diet, while the remaining population consumed a mixed diet. Most vegetarians were born into vegetarian families and adhered to it due to religious beliefs. Additionally, some individuals converted to vegetarianism for perceived health benefits, such as efficient weight management and decreased cholesterol levels. However, many vegetarians reported deficiencies in certain micro and macronutrients, necessitating additional supplementation. Lacto-ovo vegetarians were found to experience the most health benefits from this practice, exhibiting desirable body mass index rates and higher rates of satisfaction.

Conclusions

Vegetarianism is prevalent in the urban population of Hyderabad, India. The decision to follow a vegetarian lifestyle is influenced by various factors, including religious beliefs and perceived health benefits. However, it is important to address the potential nutrient deficiencies associated with vegetarian diets. Lacto-ovo vegetarians, in particular, seem to derive significant health benefits from their dietary choices.

## Introduction

Vegetarianism, as defined by the Oxford Dictionary, refers to the conscious avoidance of consuming animal flesh, often including fish, and sometimes extending to animal products [1]. While it is a matter of personal preference, this practice is predominantly observed in certain Asian cultures such as individuals following Hinduism, Jainism, and Buddhism. In recent years, vegetarianism has witnessed a significant surge in popularity for a multitude of reasons [2]. The advent of the internet has facilitated its rapid adoption in the Western world, driven by perceived health benefits, including improved cardiovascular health, environmental sustainability, and enhanced animal welfare [3].

Even in nations like the United States, the United Kingdom, and Mexico, where meat and animal products form a substantial part of the diet, vegetarianism is not uncommon [4]. In India, a considerable proportion of the population, approximately 30% (10-62% in different regions), adheres to vegetarianism [4]. With the availability of meat alternatives and growing activism, the practice of vegetarianism continues to gain momentum.

Experts have debated the long-term consequences of vegetarian diets, with one primary argument being that vegetarians have a lower protein intake compared to omnivores. However, reports have indicated that while vegetarians may consume lower total protein amounts than omnivores, protein intake relative to body mass does not differ significantly between the two groups [5]. Another argument suggests that vegetarians and vegans are at a higher risk of vitamin deficiencies, particularly vitamin B12 [6]. Such deficiencies can be attributed to the biochemical composition of vegetarian dishes but can be easily addressed through the use of nutraceutical supplements.

Despite these arguments, plant-based diets have been shown to have a substantial impact on the composition and function of the gut microbiome, which, in turn, influences overall health. The EPIC-Oxford study, conducted in the 1990s, reported that vegetarians had a 32% lower risk of incident ischemic heart disease hospitalizations and deaths caused by circulatory diseases compared to non-vegetarians [7]. Another study, the Adventist Health Study 2, also revealed that vegetarianism was associated with a lower incidence of type 2 diabetes [8].

The benefits of vegetarianism extend beyond health and encompass positive environmental impacts. The adoption of nutritionally balanced vegetarian diets has been recognized as an effective strategy for reducing greenhouse gas emissions worldwide [9].

Through this cross-sectional study, we aim to analyze the knowledge and prevalence of vegetarianism in the urban population of Hyderabad, India. This study seeks to highlight the various benefits of the vegetarian way of living, along with the opinions and beliefs of vegetarians in our country. Additionally, we have recorded the challenges faced by individuals following this diet in terms of severity.

## Materials and methods

In this cross-sectional study, a total of 300 participants were recruited from a specifically selected urban area in Hyderabad, India. The selection of Hyderabad was based on its diverse population demographic, encompassing approximately 60% Hindus, 30% Muslims, and 3% Christians, allowing for a comprehensive understanding within a diverse community. Participant recruitment was conducted through an online platform, where a semi-structured questionnaire was made available to willing participants. The inclusion criteria for the study involved obtaining consent from individuals above the age of 16, while those who declined to participate were excluded. No specific sampling techniques were utilized during the participant selection process. Verbal consent was obtained from all participants after providing a clear explanation of the study objectives. To ensure privacy and confidentiality, participant identities were anonymized, and no personal contact details were collected, except for age and religion. The data collection period extended over two months from May 2018 to July 2018. The semi-structured questionnaire was self-administered by the participants. The WHO guidelines were used as the standard for evaluating body mass index (BMI). The research was conducted by a medical student under the guidance of the Department of Community Medicine, while trained researchers or interviewers were not directly involved in the data collection process. The collected data were stored using Google Forms and subsequently extracted into Microsoft Excel for analysis. The analysis was performed using both Microsoft Excel and Open Epi software. The chi-square test was employed as the primary statistical test for appropriate calculations and analysis, without the utilization of additional statistical tests. Ethical clearance for the study was obtained from the Institutional Review Board (IRB) at Gandhi Medical College (approval number: 299834) before the initiation of the study. The IRB at Gandhi Hospital, a longstanding committee existing for over a century, consists of department chairs, hospital directors, a police officer, and a legal team representative. The Dean of the institution serves as the head of the IRB. The study protocol underwent a formal review process, and upon approval by the IRB, ethical clearance was granted to ensure compliance with ethical guidelines and regulations.

## Results

Age-wise distribution

Among the study population, 74% of the individuals aged 30 or below were found to be vegetarians, whereas 26% of the population above 30 years of age were vegetarians. The findings are summarized in Table 1.

**Table 1:** Age-wise distribution (n = 300).

Age (year)	Number	%
16–30	222	74
>30	88	26

BMI-based distribution

The proportion of the study population with BMI corresponding to the normal range was found to be 60%, whereas the proportion of the underweight population was 12.6%, the overweight population was 22.6%, and obese participants constituted 4.6% of the study population.

Religion-wise distribution

In our study, 83.3% of the participants were Hindus, 6.7% were Muslims, 5% were Christians, 2% were Jains, 1.3% were Buddhists, and 1.7% were Atheists.

Study population distribution

**Figure 1:**
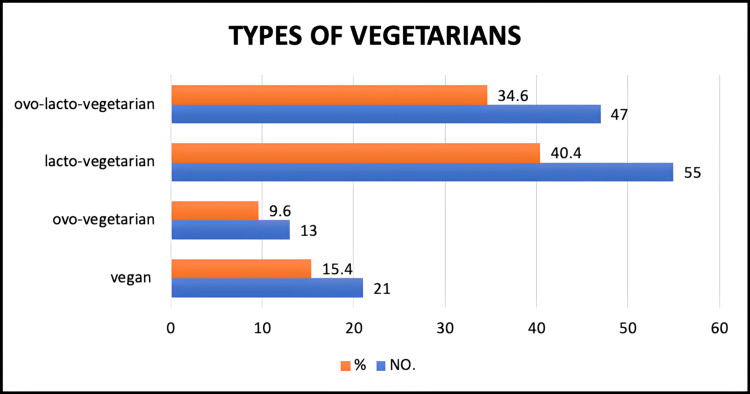
Vegetarianism types.

The study population had a distribution of 43.7% vegetarians, and the remaining 56.3% were following a non-vegetarian/mixed diet. Of the 43.7% vegetarians, 40.4% were lacto-vegetarians, 34.6% were ovo-lacto-vegetarians, 15.4% were vegans, and 9.6% were ovo-vegetarians. The findings are summarized in Figure 1.

Duration of following a vegetarian diet

Of the study population constituting of vegetarians, 53% were found to be vegetarians since birth, whereas 29.5% were vegetarians for more than five years, 11.4% were vegetarians for one to two years, and the remaining 6.1% were vegetarians for the past three to five years (Table 2).

**Table 2:** Duration of vegetarianism.

Duration	Number	%
By birth	70	53
1–2 years	15	11.4
3–5 years	8	6.1
More than 5 years	39	29.5

Reasons for conversion to vegetarianism

From the study population of individuals following a vegetarian diet, 48.7% made the conversion due to animal welfare and environmental reasons, with 31% due to religious reasons, 22.7% due to health issues, 9.2% due to latest trends and social media influence, 6.7% due to economical concerns, 4.2% due to peer pressure, 2.4% due to personal interest, 1.7% due to spiritual reasons, and 0.8% due to family pressure to follow a vegetarian diet (Table 3).

**Table 3:** Reasons for choosing a vegetarian diet.

Reasons	Number	%
Health issues	27	22.7
Peer pressure	5	4.2
Animal welfare and the environment	57	48.7
Economic concern	8	6.7
Latest trends and social media influence	11	9.2
Religious reasons	37	31
Spiritual reasons	2	1.7
Family pressure	1	0.8
Personal interest	3	2.4

**Figure 2:**
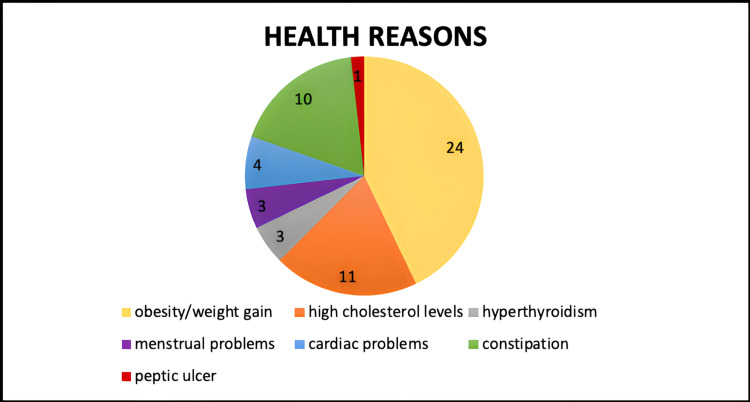
Health reasons for choosing vegetarianism.

Of the population that chose the diet due to health reasons, 44.9% did so due to obesity/weight gain issues, 29.7% due to high cholesterol levels, 27% due to constipation, 10.8% due to cardiac problems, 8.1% due to hyperthyroidism, 8.1% due to menstrual problems, and 2.7% due to peptic ulcer repercussions (Figure 2).

Awareness of the difference in diet

In our study population, the proportion of people who believed the vegetarian diet to be healthier than the non-vegetarian/mixed diet was 33%, whereas 44% of the population believed that the vegetarian diet was not healthier than the non-vegetarian diet. The remaining 23% thought that both diets were equally healthy (Table 4).

**Table 4:** Awareness of the health benefits of a vegetarian diet.

Vegetarian diet is better	Number	%
Yes	99	33
No	132	44
Equal to other diets	69	23

**Figure 3:**
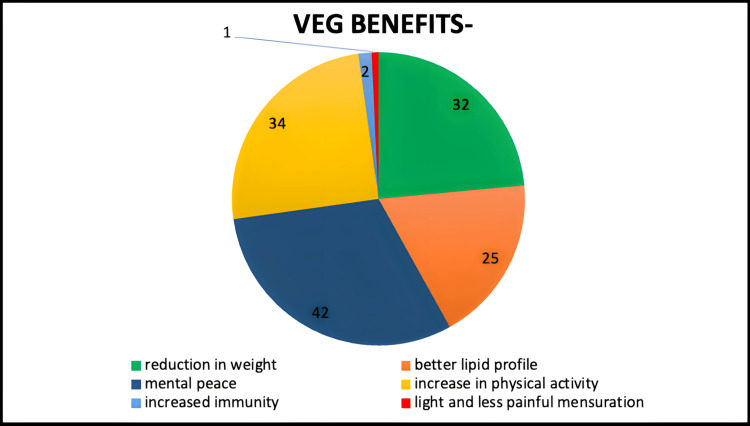
Health benefits associated with vegetarianism.

The health issues alleviated by following the vegetarian diet were found to be a reduction in weight in 39.5%, mental peace in 51.9%, better lipid profile in 30.9%, increase in physical activity in 42%, increased immunity in 2.5%, and light and less painful menstruation in 1.2% of the study population (Figure 3).

Of the population, 34.9% were aware of the health benefits associated with vegetarianism, whereas the remaining 65.1% were unaware.

Dietary supplementation in the study population

**Figure 4:**
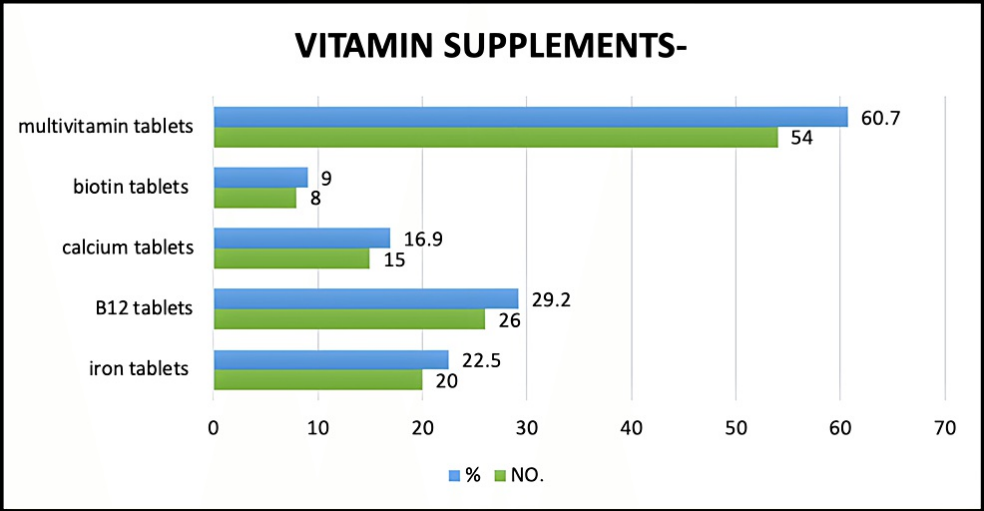
Vitamin supplements taken by vegetarians.

In the study population, 29.2% took single or multiple vitamins and other dietary supplements in addition to their normal diet, whereas 70.8% took some supplements. In this study, 60.7% admitted to taking multivitamin tablets, 29.2% took vitamin B12 tablets, 22.5% took iron tablets, 16.9% took calcium tablets, and 9% took biotin tablets (Figure 4).

Of the population consuming dietary supplementations, 76.5% did so on recommendation by a doctor, 20% on advice from family and friends, 9.4% did so due to research from sources on the internet, and 4.7% self-medicated (Table 5).

**Table 5:** Reasons for vegetarians taking supplements.

Reasons	Number	%
Advice from family and friends	17	20
On doctors recommendation	65	76.5
From sources on the internet	8	9.4
Self	4	4.7

Dietary imbalances in the study population

**Figure 5:**
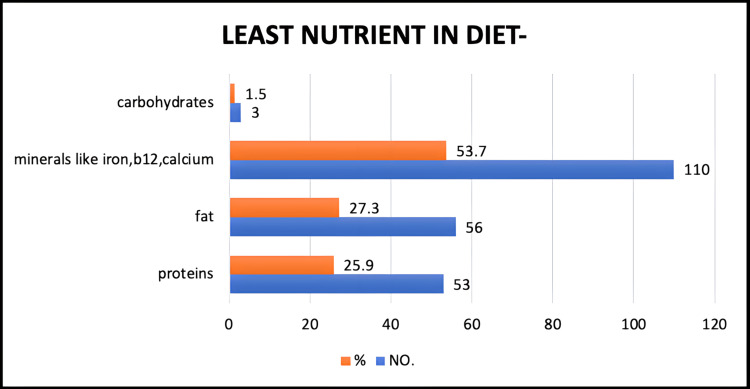
Least found nutrient in the diet.

In the population, 53.7% were found to have the least amounts of minerals such as iron, B12, and calcium in their diet, whereas 27.3% had the least amounts of fats, 25.9% had the least amounts of proteins, and the remaining 1.5% were found to have the least amounts of carbohydrates in their respective diets (Figure 5).

Of the vegetarians, 31.6% of the population had difficulty maintaining a balanced diet with their current diet, while the remaining 68.4% had no difficulty maintaining a balanced diet.

**Figure 6:**
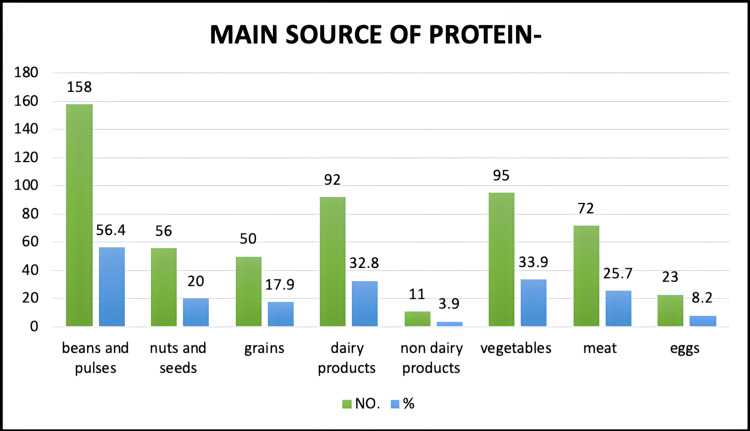
Main protein source.

In the study population, 56.4% had beans and pulses as their main source of protein, 25.7% had meat as their main source of protein, 33.9% had vegetables, 32.8% had dairy products, 20% had nuts and seeds, 17.9% had grains, 8.2% had eggs, and the remaining 3.9% had non-dairy products as the main source in their diet (Figure 6).

Awareness about a vegetarian diet

In the study population, 40.3% knew a moderate amount about the vegetarian diet, 26.3% knew very little, 14.3% knew quite a lot, 10% knew a lot, and the remaining 9% knew nothing about a vegetarian diet and lifestyle.

Limitations of a vegetarian diet

**Figure 7:**
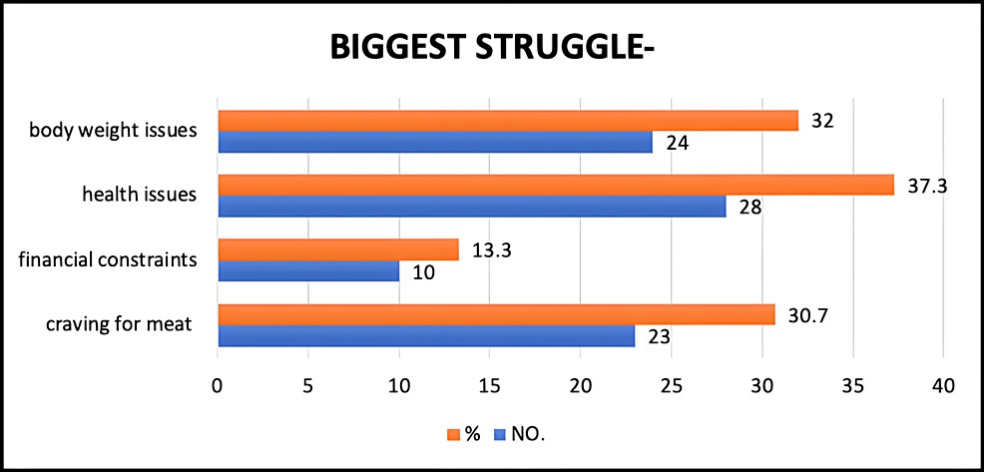
Biggest struggle while following the diet.

In our study population of vegetarians, 30.7% had a craving for meat as their biggest struggle in continuing the vegetarian diet, 37.3% had health issues, 32% had body weight issues, and the remaining 13.3% had financial constraints as their biggest struggles to continue with their present vegetarian diet (Figure 7).

In our study population of vegetarians, 52.2% reported vitamin deficiencies, 34.8% had protein deficiency, 21.7% had anemia, 2.2% had depression, and 2.2% had developed weakness (Table 6).

**Table 6:** Main health issue developed post-dietary change.

Health issue	Number	%
Anemia	10	21.7
Protein deficiency	16	34.8
Vitamin deficiency	24	52.2
Depression	1	2.2
Weakness	1	2.2

**Figure 8:**
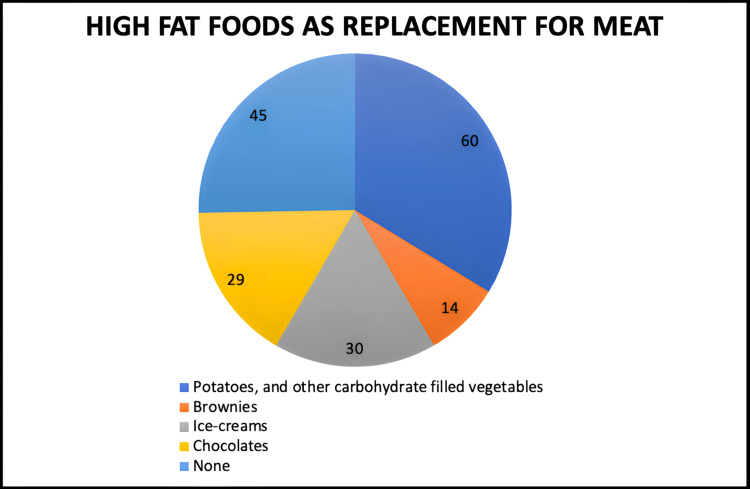
High-fat foods consumed as meat replacements.

In our vegetarian diet, 34% consumed potatoes and other carbohydrate-filled vegetables to replace meat, 17% consumed ice creams, 16% consumed chocolates, 8% consumed brownies, and the remaining 25% did not consume any high-fat foods (Figure 8).

Of the vegetarians considered, 64.4% were found to be perfectly satisfied with their diet, 15.2% were partially satisfied with their diet, 7.6% wished they were still following a non-vegetarian diet, and the remaining 12.9% had no opinion (Table 7).

**Table 7:** Level of satisfaction with a vegetarian diet.

Level of satisfaction	Number	%
No, I wish I could be non-vegetarian	10	7.6
Yes, I am partially satisfied	20	15.2
Yes, I am perfectly satisfied	85	64.4
No opinion	17	12.9

Relationship between age and vegetarianism

In our study, the number of vegetarians aged 30 years and below was found to be 76 individuals (34%), and above 30 years was 55 individuals (70%), whereas the number of non-vegetarians below 30 years of age was found to be 146 (65%), and above 30 years was 23 (35%).

The difference in vegetarians in persons aged 30 and below and in persons aged more than 30 years was found to be 36%, and the difference between non-vegetarians who belonged to the categories of age 30 years and below and those who were aged above 30 was found to be 30%, with a statistically significant p-value of 0.00000123 (chi-square = 30.8824).

Relationship between religion and the vegetarian lifestyle

In our study, the number of Hindu vegetarians was 111 (84.7%), whereas those following other religions were 20 (15.2%). The number of non-vegetarians who followed the Hindu religion was 139 (82.2%), while the number of non-vegetarians following other religions was 30 (17.7%). The difference between vegetarians who followed the Hindu religion and those who followed other religions was found to be 69.5%, while the difference between non-vegetarians who followed the Hindu religion and other religions was found to be 64.5%, with a statistically insignificant p-value of 0.56688216 (chi-square = 0.3279).

Differences in opinion about a healthier diet

In our study, the number of vegetarians who believed that the vegetarian diet was healthier or equal to other diets was 112 (85.4%), whereas those who believed that their diet was not as healthy as a non-vegetarian diet was 19 (14.5%). Non-vegetarians who believed that the vegetarian diet was better or equal were 56 (33.1%), while the rest 113 (66.8%) believed that it was not equal to a non-vegetarian/mixed diet. The difference between vegetarians who believed that a vegetarian diet was equal or healthier when compared to other diets and those who believed that a vegetarian diet was less healthy when compared to other diets was 70.9%, while the difference between non-vegetarians who believed that their diet was equally healthy or healthier and those who believed it to be less healthy was found to be 33.7%. The finding was statistically significant with a p-value of <0.001 (chi-square = 82.101).

Reasons for conversion to a vegetarian diet

In our study, the number of vegetarians by birth who choose a vegetarian diet due to religious reasons was 34 (49.2%), while the number of those who opted for the diet due to other reasons was 35 (50.7%). The number of converted vegetarians who choose the diet due to religious reasons was 0%, while those who chose the diet due to other reasons was 71 (100%). The difference between the number of by-birth vegetarians who choose their diet due to religious reasons and other reasons was 1.5%, whereas the difference between converted vegetarians who chose the diet due to religious reasons and other reasons was 100%. The finding was statistically significant with a p-value of <0.001 (chi-square = 46.2073).

Relationship between dietary supplements and diet

In this study, the proportion of vegetarians who consumed dietary supplements was 33.5%, while those who did not was 66.4%. While the proportion of non-vegetarians who took dietary supplements was 26.6%, those who did not was 73.3%.

The difference between vegetarians who consumed dietary supplements and those who did not was 32.9%, while that between non-vegetarians who took dietary supplements and those who did not was 46.7%. The finding was statistically insignificant with a p-value of 0.19052673 (chi-square = 1.7135).

Relationship between diet and the least obtained nutrient

In our study, the number of vegetarians who obtained the least amount of minerals in their diet was 46 (38.3%), while those who got the least amounts of other nutrients was 74 (61.6%). The number of non-vegetarians who obtained the least amounts of minerals in their diet was 63 (62.3%), while those who opted for others was 38 (37.6%).

The difference between vegetarians who had minerals as the least obtained nutrient and those who had other nutrients as their least obtained nutrient in their diet was 23.3%, while the difference between non-vegetarians who obtained minerals and those who obtained other nutrients as their least obtained nutrient in their diet was 24.7%. The finding was statistically significant with a p-value of 0.00037015 (chi-square = 12.6831).

Difference between opinions about PETA and diet

In our study, the number of vegetarians who supported PETA was 105 (80.1%), and those who did not support the organization or who had no opinion about it was 26 (19.8%). The number of non-vegetarians who supported the organization was 112 (66.2%), while those who did not support it were 578 (33.7%).

The difference between vegetarians who supported PETA and those who did not or had no opinion was 60.3%, while the difference between non-vegetarians who supported PETA and those who had no opinion or did not support the organization was 32.7%. The finding was statistically significant with a p-value of 0.00768903 (chi-square = 7.1048).

Relationship between diet and the main source of protein

In our study, the number of vegetarians who consumed plant products was 220 (81.7%), and the number of vegetarians who consumed animal products was 49 (18.2%). The number of non-vegetarians who consumed plant products was 149 (51.9%), and those who consumed animal products was 138 (48%).

The difference between vegetarians who consumed plant products and those who consumed animal products as their main source of protein was 63.5%, while the difference between the number of non-vegetarians who consumed plant products and those who consumed animal products was 3.9%. The finding was statistically significant with a p-value <0.001 (chi-square = 55.495).

Relationship between satisfaction with the vegetarian diet and the duration of vegetarianism

The study showed 56 (82.3%) by-birth vegetarians were partially and perfectly satisfied, and 13 (19.1%) by-birth vegetarians were not satisfied or had no opinion about their diet. The number of converted vegetarians who were partially and perfectly satisfied with their diet was 50 (80.6%) and those who were not satisfied with their diet or had no opinion was 12 (19.3%). The difference between the number of by-birth vegetarians who were partially and perfectly satisfied with their diet and those who were not satisfied or had no opinion was 63.2%. While the difference between the number of converted vegetarians who were partially and perfectly satisfied with their diet with those who were not satisfied or had no opinion was 61.3%. The finding was statistically insignificant with a p-value of 0.9726592 (chi-square = 0.0012).

Relationship between diet and BMI

The study conducted showed that 15 (11.4%) vegetarians had a BMI that was less than or equal to 18.5 kg/m^2^, whereas 116 (88.5%) vegetarians had a BMI above 18.5 kg/m^2^. In the non-vegetarian population, 23 (13.6%) had a BMI less than or equal to 18.5 kg/m^2^, while 146 (86.4%) had a BMI greater than 18.5 kg/m^2^.

The difference between vegetarians who had a BMI less than or equal to 18.5 kg/m^2^ and those who had a BMI greater than 18.5 kg/m^2^ was 77.1%. The difference between non-vegetarians who had a BMI less than or equal to 18.5 kg/m^2^ and those with a BMI greater than 18.5 kg/m^2^ was 72.8%. The finding was statistically insignificant with a p-value of 0.57707919 (chi-square = 0.311).

## Discussion

Vegetarianism has gained popularity as a dietary choice among many individuals in recent times. Epidemiological studies have shown that vegetarians tend to have better BMI and experience several health benefits compared to non-vegetarians, including lower cholesterol levels, reduced risk of hypothyroidism, and less painful menstruation [10]. In our study, 43.7% of the participants were vegetarians, with 53% being vegetarians from birth and the rest converting to vegetarianism. The main reasons for conversion included obesity/weight gain (64.9%), high cholesterol levels (29.7%), and cardiac problems (10.8%). Among these individuals, 56.49% experienced health improvements after changing their diet, suggesting that vegetarianism is associated with positive health outcomes. Our findings align with those of Agrawal et al. [11], who reported that the prevalence of diabetes varied among individuals consuming different types of vegetarian diets, with the lowest rates observed in those following a lacto-vegetarian, lacto-ovo-vegetarian, or semi-vegetarian diet compared to pesco-vegetarian diets. Furthermore, consumption of a lacto-vegetarian, lacto-ovo-vegetarian, or semi-vegetarian diet was associated with a lower likelihood of diabetes compared to a non-vegetarian diet in adjusted analyses.

Continuing from the previous discussion, our results indicated that 61.83% of vegetarians in the study population had a normal BMI, slightly higher than the 58.58% observed among non-vegetarians. Although the difference is not substantial, it is noteworthy. A study by Key et al. reported that vegetarians had a lower BMI by approximately 1 kg/m^2^ and a lower risk of diverticular disease, gallstones, and appendicitis, which is consistent with the findings of this study [12].

In addition to assessing the prevalence of vegetarian diets, our study aimed to evaluate the awareness regarding vegetarianism among the study population. The results revealed that 33% of the participants believed that a vegetarian diet was healthier than a non-vegetarian/mixed diet, while 44% believed it was not healthier, and 23% believed both diets were equally healthy. This belief can be attributed to the diverse religious demographics in the city of Hyderabad. According to the population census published by the Government of India in 2011 [13], 64.93% of the population identified as Hindus, 30.13% as Muslims, and 2.75% as Christians. Based on this data, it can be inferred that the belief that non-vegetarian diets are superior to vegetarian diets is influenced by religious practices. This inference is supported by research on the relationship between religion and food, which revealed that while most Muslims and Christians follow a meat-based diet with certain restrictions, nearly 20% of Hindus frequently consume meat [14].

Given the focus of this paper on vegetarianism in India, it is crucial to comment on the typical nutritional and dietary profile of the average Indian vegetarian, commonly referred to as the Hindu vegetarian. A study published in 2001 also supports our observations [15]. India is a country with over 28 diverse regional cuisines, each with its own indigenous dietary practices. The majority of the population consumes a variety of grain-based diets. In northern regions, staples such as wheat, barley, millet, and corn are commonly consumed, while rice is predominant in the southern regions. These grains serve as the main source of carbohydrates. Dried legumes/lentils, known as dals, are prepared in almost all households and provide a significant portion of protein and carbohydrates. Most recipes also incorporate green leafy vegetables, other cooked or raw vegetables, and oils, which are rich in micronutrients, minerals, fiber, and healthy fats. Curds and milk are also part of the customary diet. When all these components are consumed together, the individual micronutrients and macronutrients are typically met.

It is important to address the lesser-known drawbacks of vegetarianism. Not all vegetarian practices lead to nutritional deficiencies, but certain communities that completely avoid animal products such as eggs and dairy are at a higher risk. In our study, 78.7% of vegetarians faced nutritional deficiencies and required dietary supplements such as iron tablets (22.86%), vitamin B12 tablets (25.71%), calcium tablets (8.57%), biotin tablets (5.71%), and multivitamin supplementation (37.14%). Similar findings were reported in a study from India [16]. Another study published in 2014 [4] also highlighted the potential nutritional risks associated with protein and micronutrient deficiencies in vegetarian diets. Vitamin B12 deficiency, a common concern among both Indian and Western vegetarians, has been consistently observed. Additionally, a study [10] reported that vegetarians tend to have lower skeletal muscle mass, which increases the risk of osteoporosis and fractures, especially in post-menopausal women. This could be attributed to lower vitamin D levels and overall protein intake among vegetarians.

Study limitations

Our study population was limited to 300 participants and only those above the age of 16 years were allowed to participate. Further information is needed to evaluate the statistics for Hyderabad, Telangana as a whole with extensive studies considering the entire population for confirming the study findings.

## Conclusions

Based on the study findings, several relevant conclusions can be drawn. Vegetarianism has gained popularity and is associated with various health benefits, including improved BMI, lower cholesterol levels, reduced risk of hypothyroidism, and less painful menstruation. Conversion to vegetarianism can lead to positive health outcomes, with individuals experiencing benefits such as weight loss, improved cholesterol levels, and better management of cardiac problems. Vegetarian diets are associated with a lower risk of diabetes compared to non-vegetarian diets, particularly lacto-vegetarian, lacto-ovo-vegetarian, and semi-vegetarian diets. Vegetarians in the study population had a slightly higher proportion of individuals with normal BMI compared to non-vegetarians, supporting the notion that vegetarianism may contribute to maintaining a healthy body weight.

Awareness regarding the healthiness of vegetarian diets varied among the study population, with some participants perceiving vegetarian diets as healthier, while others did not share the same belief. This perception was influenced by religious practices and demographics. The typical dietary profile of Indian vegetarians, characterized by a variety of grains, legumes, vegetables, and dairy products, provides a balanced mix of macronutrients and micronutrients. While vegetarianism offers many benefits, it is essential to address potential nutritional deficiencies, particularly among communities that completely avoid animal products. Supplementation with vitamins and minerals, such as iron, vitamin B12, and calcium, may be necessary to overcome these deficiencies. It is crucial to recognize the potential risks of protein and micronutrient deficiencies in vegetarian diets and take appropriate measures to ensure a well-rounded and balanced nutrient intake. Overall, the findings highlight the positive impact of vegetarianism on health outcomes while emphasizing the importance of addressing nutritional needs to maintain optimal well-being.

## Appendices

Questionnaire:

Age: ____years

Sex: M F others

Occupation: __________________

Weight: ___kg

Height: ___cm

Religion: Muslim, Hindu, Christian, Jain, Buddhist, and Others

Total family income: ___________INR/month

1. How would you describe your diet?

Vegetarian         Mixed

2. To which category do you belong?

Vegan (only vegetables)

Ovo-vegetarian (vegetables+eggs)

Lacto-vegetarian (vegetables+dairy and dairy products)

Ovo-lacto-vegetarian (vegetables+eggs+dairy and dairy products)

3. Since when have you been a vegetarian?

By birth

1-2 years

3-5 years

More than 5 years

4. What is/are your reason(s) for converting into a vegetarian?

Health issues

Peer pressure

Animal welfare and the environment

Economical concern

Latest trends and social media influence

5. What are your health reasons for becoming a vegetarian? (If any then specify)

Obesity/weight gain

High cholesterol levels

Hyperthyroidism

Menstrual problems

Cardiac problems

Constipation

Others, ________________

6. Do you think a vegetarian diet is healthier than a non-vegetarian diet?

Yes        No         Equal to other diets

7. Are you aware of any health benefits that being vegetarian can cause?

Yes        No

8. Have you personally noticed any health benefits after converting to vegetarianism?

Yes        No

9. If yes, the benefits were:

Reduction in weight

Better lipid profile

Mental peace

Increase in physical activity

Others, ________________________________________

10. Do you think you can get sufficient amounts of vitamins and nutrients with a vegetarian diet?

Yes        No

11. Do you take any vitamin or mineral supplements?

Yes        No

12. If yes, what are the supplements you take?

Iron tablets

B12 tablets

Calcium tablets

Biotin tablets

Multivitamin tablets

13. On whose recommendation did you start taking the supplements?

Advice from friends and family

On the doctor’s recommendation

From sources on the internet

Others, _______________________

14. Which of the following do you get the least of in your diet?

Protein

Fat

Minerals like iron, B12, and calcium.

Others, ________________

15. Do you find it difficult to have a balanced diet as a vegetarian?

Yes        No

16. Which of the following is the main source of protein in your diet?

Beans and pulses

Nuts and seeds

Grains

Dairy products

Non-dairy products (soya milk, vegan cheese)

Vegetables

Others, ______________________

17. Did you research the vegetarian diet before making the conversion to it?

Yes        No

18. How much do you know about the vegan/vegetarian diet?

Nothing

Very little

Moderate amount

Quite a lot

A lot

19. What would be your biggest struggle if you had to continue being a vegetarian/vegan?

Craving for meat

Financial constraints

Health issues

Body weight issues

20. Did you develop any health issues after converting to vegetarianism?

Anemia

Protein deficiency

Vitamin deficiency

Others, ________________

21. Do you consume any of the vegetarian foods which contain high amounts of fat to replace the meat in your diet?

Potatoes and other carbohydrate-filled vegetables

Brownies

Ice-creams

Chocolates

None

22. Do you enjoy your lifestyle of eating after starting your vegetarian diet?

No, I wish I could be non-vegetarian

Yes, I am partially satisfied

Yes, I am perfectly satisfied

No opinion

23. Have you relapsed back to non-vegetarian food for any amount of time?

Yes, only on one specific occasion

Yes, frequently

No

24. Do you miss or have a craving for non-vegetarian food since starting the diet?

Yes        No

25. Is there any peer pressure involved during outings/vacations/dining out to eat non-vegetarian food?

Yes        No

26. Do you support PETA? (People for the Ethical Treatment of Animals)

Yes        No

27. If you had children would you raise them to be vegetarian/vegan?

Yes        No

28. Do you recommend the vegetarian/vegan diet to your family, friends, and others?

Yes        No
